# ABCDE cognitive aid tool in patient assessment – development and validation in a multicenter pilot simulation study

**DOI:** 10.1186/s12873-020-00390-3

**Published:** 2020-12-04

**Authors:** David Peran, Jiri Kodet, Jaroslav Pekara, Lucie Mala, Anatolij Truhlar, Patrik Christian Cmorej, Kasper Glerup Lauridsen, Ferenc Sari, Roman Sykora

**Affiliations:** 1 Prague Emergency Medical Services, Prague, Czech Republic; 2 Division of Public Health, 3rd Faculty of Medicine, Charles University in Prague, Prague, Czech Republic; 3 Secondary Nursing School and Nursing College in Prague, Prague, Czech Republic; 4 Medical College, Prague, Czech Republic; 5 Emergency Department, Motol University Hospital, Prague, Czech Republic; 6 Emergency Medical Services of the Hradec Kralove Region, Hradec Kralove, Czech Republic; 7 Faculty of Medicine in Hradec Kralove, University Hospital Hradec Kralove, Charles University in Prague, Hradec Kralove, Czech Republic; 8 Emergency Medical Services of the Usti nad Labem Region, Usti nad Labem, Czech Republic; 9 Faculty of Health Studies, Jan Evangelista Purkyne University, Usti nad Labem, Czech Republic; 10 Department of Critical Care Medicine, Children’s Hospital of Philadelphia, Philadelphia, USA; 11 Research Center for Emergency Medicine, Aarhus University Hospital, Aarhus, Denmark; 12 Department of Internal Medicine, Randers Regional Hospital, Randers, Denmark; 13 Emergency Department, Skellefteå District General Hospital, Skellefteå, Sweden; 14 Department of Anaesthesia and Intensive Care Medicine, 3rd Faculty of Medicine CU and University Hospital Kralovske Vinohrady, Charles University, Prague, Czech Republic; 15 Emergency Medical Services of Karlovy Vary Region, Karlovy Vary, Czech Republic

**Keywords:** Patient assessment, Advanced life support, Peri-arrest, ABCDE approach

## Abstract

**Background:**

The so called ABCDE approach (Airway-Breathing-Circulation-Disability-Exposure) is a golden standard of patient assessment. The efficacy of using cognitive aids (CA) in resuscitation and peri-arrest situations remains an important knowledge gap. This work aims to develop an ABCDE CA tool (CAT) and study its potential benefits in patient condition assessment.

**Methods:**

The development of the ABCDE CAT was done by 3 rounds of modified Delphi method performed by the members of the Advanced Life Support Science and Education Committee of the European Resuscitation Council.

A pilot multicentre study on 48 paramedic students performing patient assessment in pre-post cohorts (without and with the ABCDA CAT) was made in order to validate and evaluate the impact of the tool in simulated clinical scenarios.

The cumulative number and proper order of steps in clinical assessment in simulated scenarios were recorded and the time of the assessment was measured.

**Results:**

The Delphi method resulted in the ABCDE CAT. The use of ABCDE CAT was associated with more performed assessment steps (804: 868; OR = 1.17, 95% CI: 1.02 to 1.35, *p* = 0.023) which were significantly more frequently performed in proper order (220: 338; OR = 1.68, 95% CI: 1.40 to 2.02, *p* < 0.0001). The use of ABCDE CAT did not prolong the time of patient assessment.

**Conclusion:**

The cognitive aid for ABCDE assessment was developed. The use of this cognitive aid for ABCDE helps paramedics to perform more procedures, more frequently in the right order and did not prolong the patient assessment in advanced life support and peri-arrest care.

**Supplementary Information:**

The online version contains supplementary material available at 10.1186/s12873-020-00390-3.

## Background

The ABCDE assessment (Airway-Breathing-Circulation-Disability-Exposure) is the standard of care used by the European Resuscitation Council for advanced life support and many other international organisations in every patient examination. It is referred to in several parts of the European Resuscitation Council (ERC) Guidelines 2015 [1–4] and is a part of the curriculum of the ERC Advanced Life Support (ALS) Provider Course as an international standardised course [5]. The ERC Guidelines are one of the few documents that mention ABCDE on such level as international guidelines are. To the best of our knowledge there is no published ABCDE tool used as a cognitive aid (CA) so far and studies addressed this topic are lacking. The use of cognitive aids and its time consumption was also part of International Liaison Committee on Resuscitation PICOs (population, intervention, comparator, outcome) questions [6]. On the other hand there are published CAs and checklists to provide trauma care, which are in many details different from the approach to non-traumatic patients where the ABCDE approach is used. CAs are a promising means of support for resolving complex, rare or critical situations [7]. The effect of each CA should be tested and validated before implementation into the practice.

The primary and main objective of this study is to develop an ABCDE CA tool (CAT). Secondary objective is to examine whether the CAT can improve patient assessment using ABCDE approach in number and order of assessments performed and shortening examination time.

## Methods

### Development of ABCDE cognitive aid tool

First design of the ABCDE CAT was made by the Non-physician Section of the Czech Society of the Emergency and Disaster Medicine based on the information in the ERC Guidelines and ALS Provider Course Manual [1, 5]. This tool was offered to the ERC ALS Science and Education Committee (SEC). The ERC ALS SEC conducted a modified Delphi process with three consensus rounds including 10 members from the ERC ALS SEC started in December 2018. The processing and design of an individual CA can be evaluated by using the “Cognitive Aids in Medicine Assessment Tool (CMAT)” [8]. The potential advantages are: linear design, single page, simple typeface, minimal use of one colour base for the entire block [9]. A linear design algorithm improves teamwork more than a branched algorithm [10].

Round 1: The first round was done as an electronic survey where respondents could provide free text comments on the first design of the ABCDE CAT– regarding what should be excluded or included. Their responses were collected in 1 month via emails. During the first round of an email communication 11 suggestions for changes were collected. The survey was responded by 4 group members (of 10). Due to the low response rate an email with the suggestions for changes was sent to the other members. All of the 10 respondents agreed with 7 suggestions. Four suggestions were adopted with changes. The survey also resulted in one new suggestion.

Round 2: All comments were added to an online survey (February 2019) and all SEC members voted if they wanted to keep the suggested changes or not. New comments were also collected at this instance. At this point 7 full changes and 4 partial changes were made and the CA was sent again for final comments and suggestions. One new suggestion occurred during the survey. All changes were adopted after the survey and sent out for another round (round 3) of free text comments by email.

Round 3: A teleconference was made to discuss the last changes of content and graphics. The final approval was done after 1 month on 1st May 2019.

### Study to validate the ABCDE cognitive aid tool

#### Study design

A multicentre simulation pilot study on paramedic students performing patient assessment in pre-post cohorts design (without and with the use of ABCDE CA tool) was done in September 2019 in order to evaluate the impact of the ABCDE CA tool. The study took place on two paramedic schools in Prague (Czech Republic).

Representatives of the paramedic programmes of both schools made a written consent to participate in the study. The participation in the study was voluntary for paramedic students. All of the participants made a verbal consent to participate in the study.

The study design is in compliance with Czech legislation and ethical regulations in the Czech Republic. No ethical committee approval was needed.

#### Study location and setting

Students from two different paramedic schools in Prague participated in the study. Higher Professional School with paramedic study on the level of graduate certificate (Secondary Nursing School and Nursing College 5. kvetna, Prague, Czech Republic) with number of participants *n* = 24 and University School with paramedic bachelor degree study (Medical College, Prague, Czech Republic) with number of participants *n* = 24. The schools have different curricula but the same set of final competences of the students.

The simulation part of the study was performed in a simulation centre of the Prague Emergency Medical Services with high fidelity environment.

#### Eligibility criteria

Only students who completed their first and second year of the full-time study programme were included. Students with ongoing jobs as paramedic, emergency medical technician or nurse were excluded.

The potential bias of the students experience with ABCDE approach as part of school curricula was minimised by using two different schools with different curriculum, but the same competences after graduation, and students from different years of study. To minimise the influence of the researchers the simulations were evaluated by two observers and the results were analysed by two independent researchers.

The group of students consisted of 48 students, 46 students of them (95.8%) were familiar with the ABCDE approach before the study but 0 (0%) had specific training in the ABCDE approach.

#### Interventions

All study participants performed patient assessment alone without any further help on scene. There was an instructor of the scenario and the patient was impersonated by an experienced paramedic responding realistically according to the scenario (standardized patient). The assessment findings were as presented by the actor and if some invasive examination was needed the lead instructor provided the results. All standard equipment of an emergency department was on a trolley. In scenario 2 (described later) the participants got the ABCDE cognitive aid at the beginning of the simulation without any other preparation or instructions on how to use it.

Each participant did two similar scenarios (first without and then with the ABCDE CA tool) with at least 1 h break between the scenarios. The descriptions of the scenarios are shown below. During 5 min of briefing the participants were asked to perform a proper A-to-E assessment during the simulation, without pointing out the endpoints of the study.

**Simulated scenario 1** (without the ABCDE cognitive aid). **Situation:** A gentlemen (born 1945) brought by his wife to the emergency department of the university hospital. He hasn’t been feeling well since yesterday, he is weak and slightly confused. You are asked to perform an examination on Emergency Department (ED) including all diagnostic aids.

**Simulated scenario 2** (with the ABCDE cognitive aid). **Situation:** A gentlemen (born 1950) coming to the emergency department of the university hospital. He hasn’t been feeling well since yesterday and feels weakness of his legs. You are asked to perform an examination on ED including all diagnostic aids.

#### Outcome measures

There was a total of 1584 potential assessment steps to achieve during each scenario by all participants (each scenario has 33 assessment steps, multiplied by number of participating paramedic students). The time needed to resolve the scenarios was tested by the ALS instructors and identically determined for both scenarios to be 5 min.

**Fig. 1:**
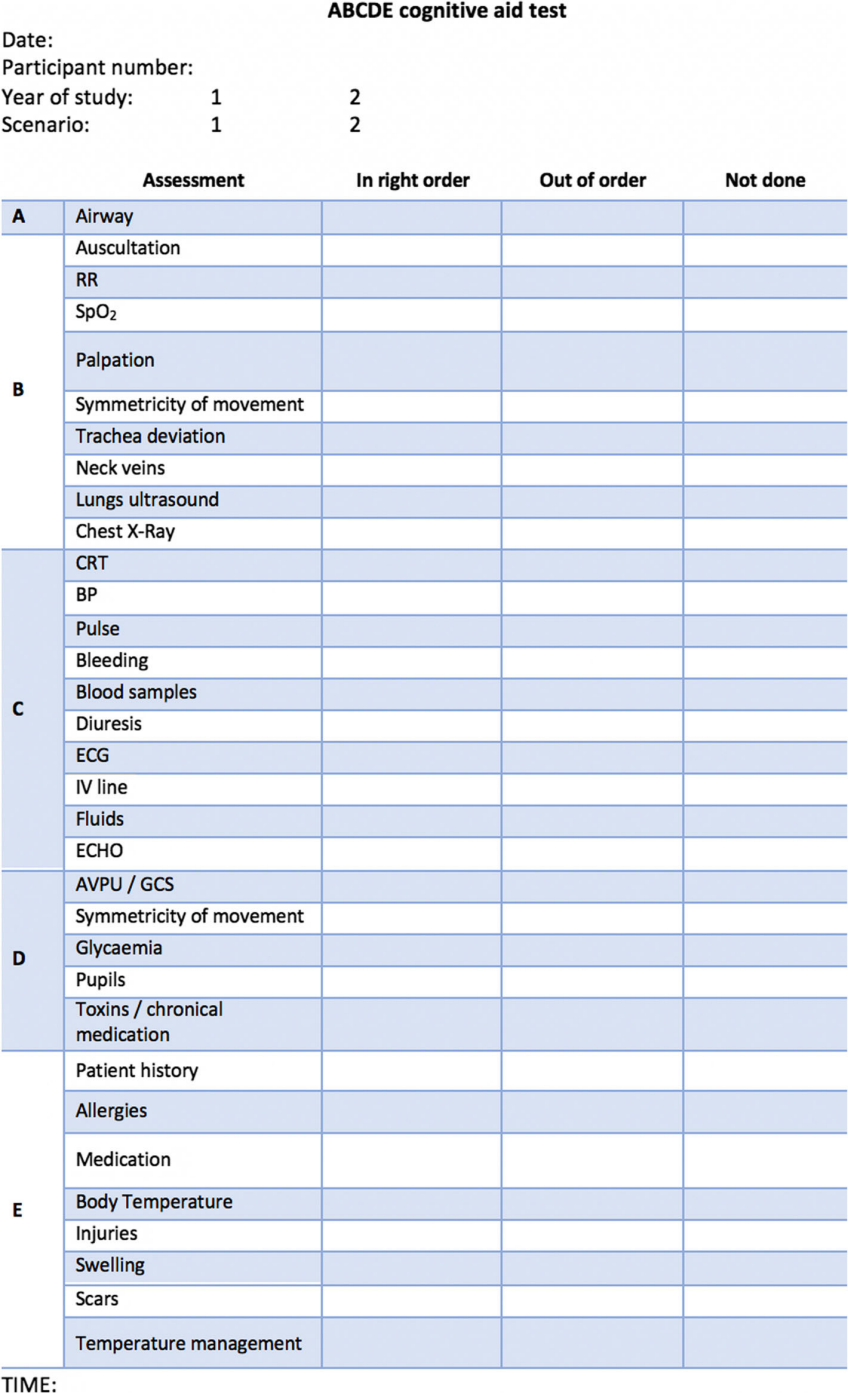
Evaluation form. Legend: RR – respiratory rate; CRT – capillary refill time; BP – blood pressure; ECG – electrocardiogram; IV – intravenous; AVPU – Alert/Voice/Pain/Unresponsive; GCS – Glasgow Coma Scale

Two instructors recorded the results of each paramedic’s assessment of the standardized patients in the scenarios using evaluation forms with three columns (Fig. 1). The participant’s steps in patient assessment were evaluated as: “Made in the right order”, “Made in wrong order” and “Not made at all”. Only fully and correctly performed assessments and interventions were evaluated as complete. Instructors also measured the time needed to complete the A-to-E assessment.

#### Sample size and data analysis

This is pilot study with no available data from previous studies. The sample size was not determined and calculated before starting the study. The cohort of paramedics was chosen and defined on a voluntary basis from cooperating schools.

#### Statistical methods

Odds ratios were used to compare the proportions of correctly and timely performed steps in patient assessment with and without ABCDE cognitive aid tool. The duration of the assessment with and without CA was tested by Mann-Whitney U test after exclusion of normal data distribution by Kolmogorov-Smirnov Test. Data are presented as median and 25^th^ a 75^th^ percentile. Statistical software *STATISTICA* 7.0 (StatSoft, Inc., Tulsa, Oklahoma, USA) was used for statistical analysis, calculations and creating graphs. The significance level was stated as *p* < 0.05.

## Results

### Primary objective, the ABCDE cognitive aid tool development

**Fig. 2:**
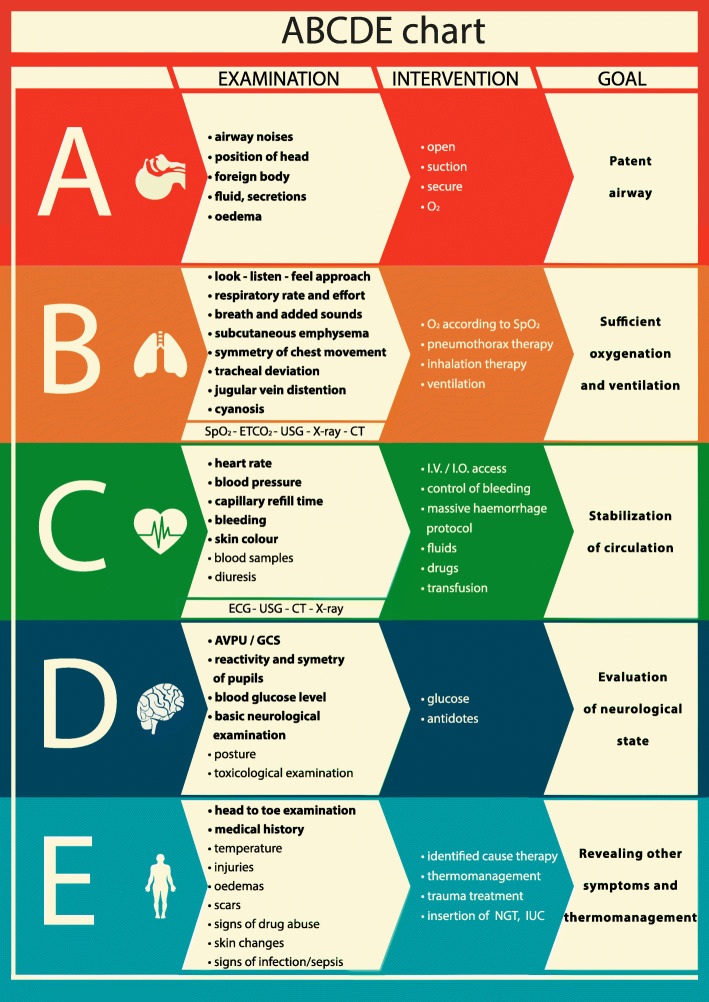
ABCDE tool

The final design of the ABCDE CA tool made during the Delphi method is shown in Fig. 2. The main aim of the tool is to visualise the assessment measures in primary and secondary assessment and to set the goal of each part (A-B-C-D-E).

### Validation pilot study results

**Fig. 3:**
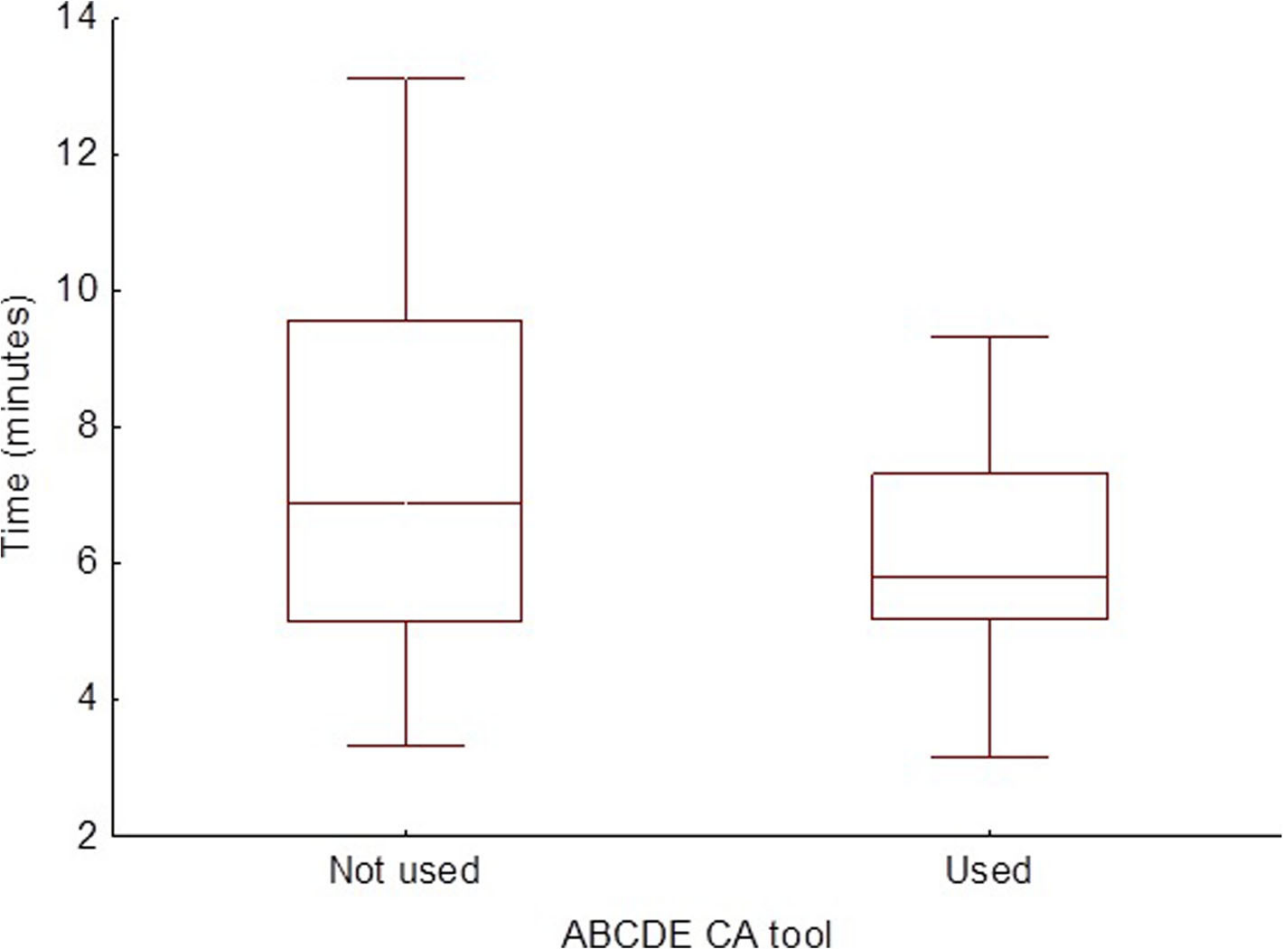
Graph of time needed to perform ABCDE evaluation. Legend: Data in box plots with whiskers are median, quartiles and range

The participant characteristics are presented in Table 1. The use of the ABCDE CAT was associated with more performed assessment steps (804 steps without and 868 with the CAT; OR = 1.17, 95% CI: 1.02 to 1.35, *p* = 0.02) which were significantly more frequently performed in proper order (220 without and 338 with the CAT; OR = 1.68, 95% CI: 1.40 to 2.02, *p* < 0.001) (Table 2). The use of ABCDE CA did not shorten or prolong the median time of patient assessment significantly (6.88 [5.17; 9.56] minutes) vs (5.79 [5.19; 7.32] minutes); (U = 921, Z = − 1.69, *p* = 0.09, *r* = − 0.018) (Fig. 3).

**Table 1:** Participant characteristics

	***N*** = 48
Age	21 (SD 2.02)
Female	24 (50%)
First year completed	20 (41.2%)
Second year completed	28 (58.3%)
Prior knowledge of ABCDE assessment	46 (95.8%)
Prior training in ABCDE	0 (0%)

**Table 2:** Objective measurements

	ABCDE CA tool not provided n (%)	ABCDE CA tool provided n (%)	
Cumulative assessments expected to be performed by paramedics in both scenarios (n, %)	1584 (100%)	1584 (100%)	NS
Assessments performed	804 (50.76%)	868 (54.80%)	P < 0.05
Assessments performed in the right order	220 (13.89%)	338 (21.34%)	P < 0.0001

## Discussion

The main finding of the study is that the newly developed ABCDE cognitive aid (CA) tool can be used to facilitate patient assessment. When used, participants provide more diagnostic steps and also more steps in the right order without consuming more time, but the results are not significant as this is only a pilot simulation study.

The primary objective of this study was to design a novel cognitive aid for patient assessment based on the ABCDE approach. The ABCDE CA is in compliance with the CMAT in the way of linear design, colours, single page and a very simple typeface, which might affect the use of it in clinical practice.

The use of cognitive aids in advanced life support have received special attention in the recent years [11–13]. The main objective of CAs is to support the execution of all the diagnostic and treatment steps in the right order and to improve technical and non-technical skills [14–17]. This statement was the basis for determination of the secondary objective – validation of the tool. The validation was done in a pilot design with voluntarily participating students. In the pilot design the ABCDE CA helped participants of this study to perform a proper A-to-E examination. Other studies also stated that CAs help to ensure that all treatment steps are performed in the right order. While they are no substitute for the expertise of the team members, CAs are an important tool for multidisciplinary teams [15, 18].

On the other hand, it has also been shown that cognitive aids that are not routinely used or are used only with minimal training are not well understood. In crisis situations they can be applied inappropriately, thus they may cause more uncertainty [19, 20]. Cognitive aid users often skip critical steps, deviate from recommended procedures or do not use the aids at all, despite evaluating them positively [20–22]. The results of this study support the statement that this particular CA is facilitating patient assessment. Students and young medical providers might derive a greater benefit from the use of CA [7]. Other studies confirm that CA is often used by younger groups as well as the most experienced providers. They are used least by healthcare providers with 2–10 years’ experience [12]. In this study the participants were all at the beginning of their professional career and the results support the claim that the ABCDE CA helps young providers.

Gilfoyle E et al. [6] published a systematic review on Cognitive Aids in Resuscitation where is mentioned that CA might prolong some procedures during resuscitation. In this study with limited number of participants the time needed to provide proper simulated patient assessment was not prolonged. Another study with greater population is needed to find out the effect of this particular tool on time consumption.

Studies also stated that CA increase the safety of provided care [23–25]. Similarly, there is the parallel of our work with research focused on the impact of the Pre-Hospital Trauma Life Support training program on improving the quality of documentation in trauma [26]. This CA seems to be a promising tool to facilitate patient assessment in education and also in clinical practice. The safety of care might be increased with clinicians providing more diagnostic steps and also more steps in the right order. Students might benefit from use of the tool when learning patient assessment in the early stage of the study process.

This pilot study brings another hypothesis for further research. A randomised controlled trial is needed to verify the results of this pilot study. More participants are needed to prove the effect on time consumption.

### Limitations

As this is a pilot study, we agree that a larger group of participants, randomisation and blinded design will be needed for more accurate results. This is also a simulation study without a validated assessment protocol. Authors used the students as participants to minimise the impact of personal professional experience of working paramedics, but the pre-screening of the students’ knowledge was done by a questionnaire only. This was an observational study, and the learning effect from the first simulation to the second cannot be excluded either. This study aimed at mastering all the steps of the ABCDE approach and they were given the same weight as this CA is not aiming on improvement of the quality of each step. We also did not evaluate any differences between the two schools as their students are comparable.

## Conclusion

This newly developed ABCDE cognitive aid chart could be used as a cognitive aid during patient assessment. The use of this cognitive aid is associated with more procedures being performed and in the right order during patient assessment provided by students. The use of cognitive aid did not prolong the examination time.

## Supplementary Information

**Additional file 1.** Description of Scenario 1 and Scenario 2.

## Data Availability

Data set is available on a reasonable request from the corresponding author.

## Abbreviations

ALS: Advanced Life Support
CA: Cognitive aid
CAT: Cognitive aid tool
CMAT: Cognitive Aids in Medicine Assessment Tool
ECG: Electrocardiogram
ED: Emergency department
ERC: European Resuscitation Council
ILCOR: International Liaison Committee on Resuscitation
OR: Odds ratio
PICO: Population, intervention, comparator, outcome
SEC: Science and Education Committee
