# Supplementary material for: ABCDE cognitive aid tool in patient assessment – development and validation in a multicenter pilot simulation study
Source: BMC Emerg Med. 2020 Dec 4;20:95. doi: 10.1186/s12873-020-00390-3 (PMC7718686; doi:10.1186/s12873-020-00390-3)
Supplement: Supplementary file 1 — Additional file 1. Description of Scenario 1 and Scenario 2. [file 12873_2020_390_MOESM1_ESM.pdf]

Simulated scenario 1 (without the ABCDE cognitive aid). **Situation:** A gentlemen (born 1945) brought by his wife to the emergency department of the university hospital. He hasn´t been feeling well since yesterday, he is weak and slightly confused. You are asked to perform an examination on Emergency Department (ED) including all diagnostic aids. Steps that are not within your competence can at least be requested or consulted with your doctor. **Background:** 6 years after myocardial infarction, high blood pressure, ischemic heart disease. Retired, lives with his wife. Medication: antihypertensives, diuretics, statin. Allergies: none. **Assessment:** A – patent, no secretion, no swelling; B – eupnoeic, respiratory rate 16', breathing sounds clear on both sides, oxygen saturation 91%; C – capillary refill time 2'', blood pressure 120/80 mmHg, palpable central and peripheral pulses, heart rate 72/min, ECG: electric activity present, rate 72', regular, QRS 80 ms, P waves present, P:QRS = 1:1, PQ 230 ms, no ischemic changes; blood samples can be requested, results will be in 10', X-ray and sonography can be requested, permanent urinary catheter if necessary, diuresis of 300 ml of light urine in the bag after insertion (fluid intake of about 500 ml today); D – alert, slightly confused, Glasgow Coma Scale 4-4-6, blood glucose 4.2 mmol/l, general weakness, symmetrical movement; E – slight swelling of the lower limbs, no injuries or other findings on the skin, temperature 36.8°C, negates other problems. **Recommendation**: Please give us a quick assessment of the patient’s condition.

Simulated scenario 2 (with the ABCDE cognitive aid). **Situation:** A gentlemen (born 1950) coming to the emergency department of the university hospital. He hasn´t been feeling well since yesterday and feels weakness of his legs. You are asked to perform an examination on ED including all diagnostic aids. Steps that are not within your competence can at least be requested or consulted with your doctor. **Background:** Patient history: past myocardial infarction, high blood pressure. Early retirement, lives with his wife. Medication: antihypertensives, diuretics, statin. Allergies: pollen, dust. **Assessment:** A – patent, no secretion, no swelling; B – eupnoeic, respiratory rate 12', breathing sounds clear on both sides, oxygen saturation 95%; C – capillary refill time 2", blood pressure 140/70 mmHg, palpable central and peripheral pulses, heart rate 65/min, electrocardiogram: electric activity present, rate 65', regular, QRS 85 ms, P waves present, P: QRS = 1: 1, PQ 180 ms, no ischemic changes; blood samples can be requested, results will be in 10', X-ray and sonography can be requested, permanent urinary catheter if necessary, diuresis of 200 ml of pale urine in the bag after insertion (fluid intake of about 700 ml today); D – alert, Glasgow Coma Scale 4-5-6, blood glucose 4.8 mmol/l, general weakness of the lower legs, symmetrical movement; E – swelling of the lower limbs, without injury, temperature 36.6°C, intact skin, negates other problems.
